# The nature of inappropriate referrals to wellness services in primary care setting in Qatar: Outcome of multifaced interventions on rate and quality of referrals

**DOI:** 10.1016/j.heliyon.2024.e31356

**Published:** 2024-05-16

**Authors:** Sarah Musa, Sami Abdeen

**Affiliations:** a Department of Preventative Health, Primary Health Care Corporation (PHCC), Doha, Qatar; b Department of Community Medicine, Hamad Medical Corporation (HMC), Doha, Qatar

**Keywords:** Cardiovascular, Physical activity, Qatar, Referrals, Risk level, Template, Wellness

## Abstract

**Objectives:**

The objectives of this study were to explore the rate and types of inappropriate referrals to the wellness services and to assess the impact of multi-level interventions on the rate of inappropriate referrals at Rawdat Al-Khail health center in Qatar.

**Methods:**

This study employed a retrospective analysis of all referrals data to Rawdat Al-Khail wellness services extracted from the Electronic Health Records (EHRs) between July 2022 and August 2023. The monthly rates and types of inappropriate referrals were calculated. In this study, pre-post analyses were performed to evaluate the impact of two sets of interventions on reducing inappropriate referral rates. The first set involved the development and distribution of e-referral pathways training manual in September 2022. The second set, implemented in April 2023, included close monitoring the rate and types of referrals, and the initiation of feedback communication between wellness services supervisors and referring physicians for advice and corrective actions.

**Results:**

A total of 966 referrals were received during the study period, with 1:5 male-to-female ratio. Of all referrals, 34.9 % were classified as inappropriate, exhibiting considerable variations among different referring health centers. The most common reason for inappropriate referrals was due to the lack of “exercise wellness gym assessment form” (23.8 %). While interventions aimed at enhancing the referral process, they did not result in a significant overall reduction in inappropriate referral rates. However, there was a noteworthy reduction in the inappropriate referrals caused by the lack of “exercise wellness gym assessment form” observed from March to August 2023 (41 %–18 %).

**Conclusion:**

This study sheds light on the complexities of wellness services referrals, revealing a high rate of inappropriate referrals that require closer scrutiny. Despite interventions not significantly reducing the rate of these referrals, it emphasizes the need for ongoing improvement strategies. Structured, periodic interventions at higher levels are recommended to enhance referral appropriateness.

## Highlights

• Referrals to wellness services follow three pathways depending on pre-exercise risk level.
• Referrals with no filling of “exercise wellness gym assessment form” was the most common type of inappropriateness.
• Complexity of referral process, information transfer, lack of feedback system and work overload could be correlated with the inconsistent changes post interventions.
• Targeted, periodic, and face-to-face interventions at the level of organization presents new opportunities to shape future interventions.

## 1 Introduction

Referral within the health system is defined as a process that links patients from one level of health care to a same or higher level that has adequate resources (equipment, skills, drugs) to assist or take over patients' management [1]. The referral system requires effective protocols of care and thorough communication between healthcare services providers to enable continuity of care and universal health coverage [2]. A cost-effective referral process is deemed as a measure of the healthcare delivery system's overall performance [3].

Appropriateness of referrals is defined in terms of patient's condition, reason for referral and capacity of care management. The knowledge and attitude of care providers/patients, clinical conditions, and local healthcare context (waiting time, location of services, availability of slots) are factors that might influence the referral quality [4]. The correct screening of patients to fit into a clearly defined referral pathway increases the functional capacity of the referral system [5]. Inappropriate referrals may result in the inefficient utilization of resources, poorer relationships between different levels of care, patient safety concerns and reduced patient satisfaction [6].

Referral processes that employ standard templates, criteria checklists, scoring systems and assessment tools were shown to improve referral quality [7,8]. Interventions to reduce referral errors include referral guidelines, educational programs targeting primary care providers, feedback opportunities, improve communications, peer review and performance monitoring [9]. Clerical screening -done by health clerks-is also an important component to capture the inappropriate referrals earlier and act accordingly [5].

Seven wellness centers are established across 31 health centers of the Primary Health Care Corporation (PHCC) in Qatar. These wellness centers provide a 12-week physical activity program that embraces 1:1 fitness sessions, group classes, swimming pool and post-workout recovery massage. All referral orders within the primary healthcare corporations' centers in Qatar are electronically processed using the patients' EHR (Electronic Health Record). The doctor initiates the referral order by selecting “referral to Facility” and electronically fills in the required information, including the destination clinic/department, time and date, reason, and status (urgent or routine). The referral pathway system and all appointments are scheduled through the wellness center utilizing the electronic health record (EHR). Referrals to the wellness services are initiated via the family physicians or general practitioners (FP/GPs). At the time of referral, physicians must complete the “Exercise Wellness Gym Assessment Form”. This form enables the calculation of the patient's risk level, accordingly patients will be referred either directly to the wellness gym (low risk), wellness assessment (moderate risk) or the healthy lifestyle clinic (high risk). The centers accept referrals for nationals, Gulf Corporation Council citizens and PHCC staff, aged 18 years and above who have one or more lifestyle-related risk factors. Each wellness center accepts referrals from pre-defined health centers according to the geographical location. In Rawdat Al-Khail (RAK), referrals to wellness are accepted internally or externally from 9 health centers (HCs) located within the central region, namely: Airport, Um Ghuwailaina, Omar Bin Khattab, Al Wakrah, South Wakra, Al Thumama, Al Meshaf, Al Saad, and Um Seneem.

Inappropriate referrals related to risk stratification may increase the hazard of adverse exercise-related events [10]. In wellness, the risk score adopted from the American College of Sports Medicine (ACSM) is a fundamental element of the patient's evaluation and hence referral decision. Patients are classified into either mild, moderate, or high risk according to the presence and absence of known cardiovascular, pulmonary, renal, liver, or metabolic disease, the presence or absence of signs/symptoms suggestive of cardiovascular, pulmonary, renal, liver, or metabolic disease and the presence or absence of CVD risk factors. Despite the numerous benefits of a structured exercise program in lowering the risk of CVD disease and age-adjusted all-cause mortality, a major concern is the increased risk of myocardial infarction and sudden cardiac death that is sometime associated with vigorous physical activity [11]. The ACSM supports the need for preparticipation screening to identify individuals at increased risk to guide a safe and effective exercise prescription [12]. This approach assists in identifying those who are suitable to commence exercise, require further medical evaluation/clearance, those who require medically supervised exercise program and individuals with medical contraindications until those conditions have been abated or are under control.

The e-referral system within the wellness center is newly implemented in August 2021 (March 2022 in RAK HC). Challenges that might have compromised the referral quality include the lack of referral pathway policy, complexity, multi-level involvement, inconsistent feedback amongst healthcare providers, limited time of FP/GPs to carry a comprehensive assessment and inadequate training on scope of the service and eligibility criteria [2]. There has been considerable concern about the number of inappropriate referrals received at wellness which can lead to various negative outcomes affecting optimal care, safety, and staff/patients’ satisfaction [6]. The construction of an evaluation framework and collection of robust performance management data can provide insight to the size of the problem and guide for effective strategies to service development. After an extensive literature review, we discovered that studies assessing the referral process to wellness services are extremely rare both globally and locally. This highlights a significant gap that needs to be addressed. Therefore, the aim of our study was to: (i) calculate the monthly rate and types of inappropriate referrals, and (ii) to assess the impact of multi-level interventions on the rate of inappropriate referrals to the wellness center between July 2022 and August 2023 at Rawdat Al-khail health center.

## 2 Materials and methods

We conducted a retrospective analysis of referral data to RAK wellness services extracted from the EHRs from July 1, 2022 to August 31, 2023. Wellness center in RAK has an average of 1800–2300 patients’ visits monthly, with a 1:4 male to female ratio. Most of the attendees aged between 37 and 64 years and 80 % are Qatari. Any referral to the wellness gym is received by the wellness receptionists and get reviewed for eligibility and appropriateness. In this study, we assessed the effect of multi-level interventions on the rate of inappropriate referrals that were delivered at two intervals: September 2022 and April 2023.

### 2.1 Referral pathway at wellness

Referrals to the wellness services follow a rigorous pre-participation risk assessment by FP/GPs initially to determine the risk level using a validated ACSM tool [13], hence referral pathway decision. Risk stratification is based on the presence and absence of known cardiovascular, pulmonary, renal, liver, or metabolic disease, the presence or absence of signs/symptoms suggestive of cardiovascular, pulmonary, renal, liver, or metabolic disease and the presence or absence of CVD risk factors (Table 1).

**Table 1:** Referral pathway to wellness services based on ACSM preparticipation risk assessment.

Risk level	Description	Action
Mild risk	CVD risk score less than 2 and, No major risk symptoms, and No major medical problems/comorbidities.	Direct referral to wellness gym.
Moderate risk	CVD risk score from 2 to 9 m and, No major risk symptoms, and, No major medical problems/comorbidities.	Referral to wellness assessment for wellness champion review.
High risk	CVD risk score 10, and/or, One major risk symptom, and/or, Major medical problems/comorbidities.	Referral to healthy lifestyle clinic.

CVD: cardiovascular disease.

### 2.2 Variables

Referrals to the wellness services were considered inappropriate if meeting one of the following criteria: referral order with no “Exercise Wellness Gym Assessment Form” hence no ACSM assessment, mild risk with no direct referral to the wellness gym, moderate risk without wellness champion review, high risk referred directly to the wellness gym and not HLS clinic, not within the age criterion or non-Qatari/non-PHCC staff. The monthly rate of inappropriate referrals was calculated for the study period as the number of inappropriate referrals divided by the total number of referrals to the wellness services within the same period.

### 2.3 Intervention

Multiple interventions were conducted at two intervals; on September 10, 2022, a training manual was developed in collaboration between the clinical information system (CIS) and the wellness services team. This manual included the new e-referral pathways from general/family clinics to wellness services according to risk levels which aligns with ACSM guidelines. It was disseminated to all health centers users (family physicians and general practitioners) through institution emails. The content of the manual consisted of illustrated figures on how to complete the “Exercise Wellness Gym Assessment Form”, compute the risk level and select the appropriate referral pathway. On April 1, 2023, a list of wellness champions and their contact information from the 10 eligible HCs was developed, printed out and displayed at reception. Additionally, an excel sheet with key performance indicators (KPIs) was created to monitor the rates and types of inappropriate referrals. Furthermore, a feedback system was established, facilitating communication between wellness services supervisors, and referring physicians or wellness champions using emails or WhatsApp. Guidance and corrective actions were conveyed through this feedback.

The multilevel intervention strategies have offered a platform for the primary health care professionals to build communication channels, mutual learning, and to have a referral protocol made available as a reference. Description of key interventions by study phases is illustrated in Table 2.

**Table 2:** The study phases and interventions timeline.

Phase	Action
*Pre intervention* (July 1, *2022)*	Monitor KPIs (rate and types of inappropriate referrals).
*Intervention -1 (10*th *Sept 2022)*	Manual training developed by the CIS and wellness team. The manual included the new e-referral pathways according to risk level. It was sent to all HCs users.
*Intervention- 2 (April 1, 2023)*	• List of wellness champions from the 10 eligible HCs & their contact information was developed, printed out & displayed at wellness reception. • An Excel sheet that includes the rate and type of inappropriate referrals per month. The sheet was monitored by wellness supervisors. • Referral feedback through emails/WhatsApp between wellness supervisors/in charge and the referring physician/wellness champion of respective HC to advise on the corrective action. Patient is contacted at this level.
*Post-intervention (ends on Aug 30, 2023)*	Monitor KPIs (rate and types of inappropriate referrals)

KPIs: key performance indicators; CIS: clinical information system; HCs: health centers.

### 2.4 Statistical analysis

The data was analysed using IBM SPSS Statistics for Windows, Version 26.0. Armonk, NY: IBM Corp. Frequencies and percentages were employed to portray descriptive statistics for categorical variables. To assess differences in inappropriate referrals between pre- and post-interventions periods, we used Chi square test to compare proportions of different types of inappropriate referrals across these periods. Additionally, we utilized Poisson and negative binomial regression models whenever suitable to analyse differences in the weekly rates of inappropriate referrals across different timeframes. Incidence risk ratio (IRR) with 95 % confidence interval (CI) were calculated to measure the strength of these associations. Statistical significance was determined at a threshold of p < 0.05.

## 3 Results

### 3.1 Referral characteristics

**Fig. 1:**
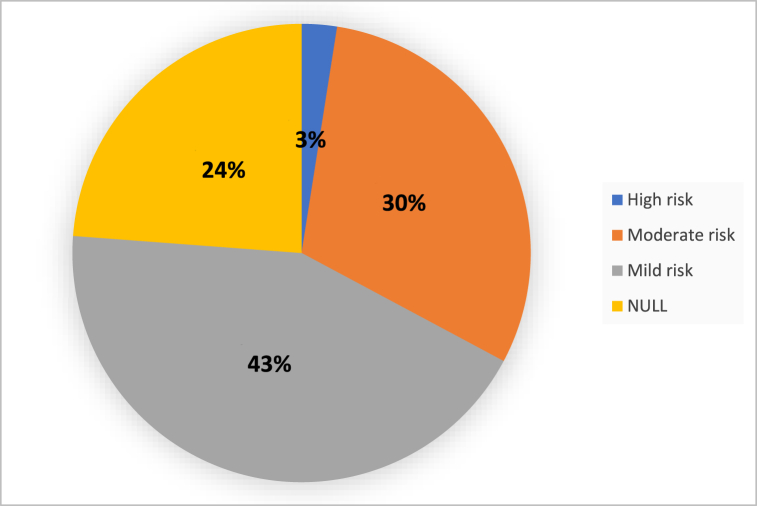
Referrals to RAK wellness services according to ACSM risk category (July 2022–August 2023, n = 966).

Table 3 illustrates the characteristics of referrals to RAK wellness services according to the referring facility from July 2022 to August 2023. During this period, 966 referrals were received with 177 male, 789 females and 21.5 % PHCC staff. The main source of referrals was from Al Thumama health center 303 (31.4 %) followed by Omar Bin Al Khatab health center 252 (26.1 %) and Rawdat Al Khail health center (internally) 131 (13.6 %). 51 patients (5.3 %) were referred from non-eligible health centers. Out of 966 total referrals, 419 (43.4 %), 293 (30.3 %) and 24 (2.5 %) were low, moderate, and high ACSM risk category respectively, while 230 (23.8 %) had no risk category computed and no “Exercise Wellness Gym Assessment Form” (Fig. 1).

**Table 3:** Referrals to RAK wellness services according to referring facility (July 2022–August 2023, n = 966).

Health Center	Total referrals		Inappropriate referrals	
	Period from July 2022–August 2023			
	NO	%	NO	%
Rawdat Al Khail	131	13.6	56	42.7
Airport	51	5.3	25	49.0
Umm Ghuwailaina	16	1.7	6	37.5
Omar Bin Khattab	252	26.1	81	32.1
Al Wakrah	29	3.0	22	75.9
South Wakrah	39	4.0	12	30.8
Al Thumama	303	31.4	72	23.8
Al Meshaf	38	3.9	10	26.3
Al Saad	20	2.1	8	40.0
Um Seneem	36	3.7	17	47.2
Other HCs, non-central	51	5.3	28	54.9
Total	966	100	337	34.8

HC: health centers.

### 3.2 Inappropriate referrals

**Fig. 2:**
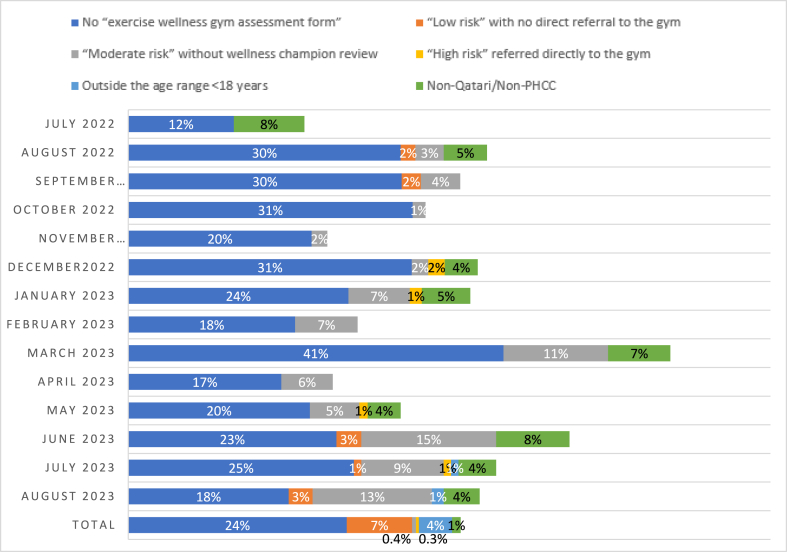
Rate of inappropriate referrals by type out of total referrals per month (July2022-August 2023, n = 966).

Out of 966 total referrals, 337 (34.9 %) were labelled as “inappropriate” A higher proportion of inappropriate referrals were observed from Al Wakrah (75.9 %), Airport (49 %) and Um Seneem (47.2 %) health centers. The most common type of inappropriate referral was those with no “Exercise Wellness Gym Assessment Form” 230 (23.8 %), followed by “moderate risk” with no referral to wellness assessment (i.e., no wellness champion review) 69 (7.1 %). Only 4 of referrals under “high risk” category were incorrectly referred directly to the wellness gym instead of the healthy lifestyle clinic, while 0.3 % were below the eligible age of 18 year. Fig. 2 and Supplementary Fig. S1 demonstrate the rate of inappropriate referrals according to the type on a monthly basis. The rate of inappropriate referrals was at highest in March 2023, 59.1 %, most of which is due to referrals with no “Exercise Wellness Gym Assessment Form”, 69.2 %.

### 3.3 Inappropriate referrals according to risk level

Among the high-risk referrals, (16.7 %) were inappropriately referred directly to the wellness gym. Additionally, among the moderate risk referrals, 26 % were inappropriate due to no wellness champion review.

### 3.4 Impact of interventions on referrals

As shown in Table 4, no observed statistically significant changes in the overall rate of inappropriate referrals between pre and post interventions. The rates of inappropriate referrals were 35.3 %, 33.6 %, and 35.8 % for pre-intervention, post first intervention and post second intervention, respectively, with P-value 0.787. However, the rate of inappropriate referrals due to lack of “Exercise Wellness Gym Assessment Form” has been reduced markedly from March to August 2023, following the second set of interventions (41 % vs. 18 %). Moreover, in comparison to the pre-intervention period, Table 5 illustrates that both post-first intervention and post-second intervention phases exhibited reduced, though not statistically significant, weekly rates of inappropriate referrals per 100 referrals (IRR 0.81, 95%CI 0.59–1.13, p = 0.218, IRR 0.84, 95%CI 0.59–1.18, p = 0.314, respectively).

**Table 4:** Distribution of different types of inappropriate referrals across intervention-phases of the study.

Types of inappropriate referrals		Pre-intervention	Post first intervention	Post second intervention	P-value^a^
**No “exercise wellness gym assessment Form”**	No	73 (71.6)	282 (73.4)	381 (79.4)	0.064
	Yes	29 (28.4)	102 (26.6)	99 (20.6)	
**Low risk with no direct referral to the gym**	No	101 (99.0)	383 (99.7)	473 (98.5)	0.174
	Yes	1 (1.0)	1 (0.3)	7 (1.5)	
**Moderate risk without wellness champion review**	No	100 (98.0)	366 (95.3)	431 (89.8)	**0.001**
	Yes	2 (2)	18 (4.7)	49 (10.2)	
**High risk referred directly to the gym**	No	102 (100.0)	382 (99.5)	478 (99.6)	>0.999
	Yes	0 (0)	2 (0.5)	2 (0.4)	
**Outside the age range**	No	102 (100.0)	384 (100.0)	477 (99.4)	0.344
	Yes	0 (0.0)	0 (0.0)	3 (0.6)	
**Non-Qatari, Non-PHCC staff**	No	97 (95.1)	375 (97.7)	459 (95.6)	0.217
	Yes	5 (4.9)	9 (2.3)	21 (4.4)	
**Inappropriate referral of any type**	No	66 (64.7)	255 (66.4)	308 (64.2)	0.787
	Yes	36 (35.3)	129 (33.6)	172 (35.8)	

a Using Chi square or Fisher exacts as appropriate.

**Table 5:** Differences in the rates of inappropriate referrals per week per 100 referrals between different phases of the study.

Stage	Rate of inappropriate referrals per week (per100 referrals)	Univariable negative binomial regression	
		IRR (95%CI)	P-value
Inappropriate referral of any type			
**Pre-intervention**	43.0	1 [Reference]	
**Post the first intervention**	35.0	0.81 (0.59–1.13)	0.218
**Post the second intervention**	36.0	0.84 (0.59–1.18)	0.314
**Inappropriate referral: No assessment form**^a^			
**Pre-intervention**	31.3	1 [Reference]	
**Post the first intervention**	28.0	0.89 (0.63–1.27)	0.529
**Post the second intervention**	21.2	0.68 (0.46–0.99)	0.043

Abbreviations: IRR, Incidence Risk Ratio.

a In the “no assessment form” part, further analysis comparing post first intervention to post second intervention showed IRR (95%CI) 1.32 (0.99–1.76) and P-value 0.060.

## 4 Discussion

In this retrospective study, we aimed to obtain data on the prevalence of inappropriate referrals to the wellness services and explore the effects of different interventions in improving the “appropriateness” of referrals. Through a process known as risk stratification, the appropriateness of referrals has been evaluated based on the following pathways: low risk with direct referral to the wellness gym; moderate risk with wellness champion review and high risk with referral to the healthy lifestyle clinic.

During the study period (July 2022 to August 23), a total of 966 referrals were received at wellness, more than three quarters of them were females. Gender disparity has been evident in physical activity interferences [1]. In the US, Craft et al. showed that females were more likely to engage in exercise and citing a preference for weight loss due to various reasons such beauty pressure, societal prospects, and time availability, when compared to males [14].

The rate of inappropriate referrals to wellness services was estimated at 34.9 % in this study. We have established a set of criteria for what is deemed inappropriate referrals considering the three attributes identified in the literature: necessity, destination, and quality of referrals [15]. Appropriateness of referrals has been also defined as the adherence to existing guidelines or predefined research criteria, provider decision making and an effective information transfer [16]. Vimalananda et al. demonstrated associations between the use of referral template embed within the EHR system and appropriateness [adjOR 1.5; 95 % CI, 1.0–2.4], clearness [adjOR 1.6; 95 % CI, 1.0–2.5]and completeness [adjOR 1.9; 95 % CI, 1.1–3.2] of referrals [17].

In our study, the most common reason of inappropriate referrals was the absence of the “Exercise Wellness Gym Assessment Form” (23.8 %), meaning “no risk level” was assigned to those referrals. Pre-exercise screening is an essential step to determine health risks and needs to ensure that the physical activity program is tailored to the patient physical capacity in a safe environment [18]. The non-automatic nature of the form in the EHR and the absence of notifications to prompt its completion at the time of referral might have contributed to our findings. High workload and demands have been linked to form incompleteness in previous literature [19,20].

In this study, a higher proportion of inappropriate referrals was observed from Al Wakrah (75.9 %), Airport (49 %) and Um Seneem (47.2 %) health centers. The detected differences in referral rates across health centers may be attributed to variations in staff training levels, being a new or old health centers, number of wellness champions and patient sociodemographic factors. Variation in age and cultural backgrounds have also been attributed to inappropriate referral due to barriers related to interaction such as language differences, poor communication skills and level of understanding [[21], [22], [23]].

The rate of inappropriate referrals due to “moderate” risk not reviewed by wellness champion was 7.1 % in this study. A situation possibly exacerbated by the complex pathway process and the requirement of multiple points involvement, from risk level identification by FP/GP, referral to wellness assessment, booking appointment at wellness assessment, and refer accordingly. This gap raises concerns as it implies that patients with moderate (or high) ACSM risk might be at risk when engaged in unsupervised or medically unfit exercise prescription [24]. Furthermore, undefined medical problems or co-morbidities such as uncontrolled diabetes mellitus, asthma, disc diseases and recent surgeries. Might pose further risk of injury or exercise-related complications if not taken into consideration. For example, diabetic individuals with no optimal blood glucose control or using insulin/insulin secretagogues should be adequately hydrated and closely monitored pre and during exercise for ketosis and hypoglycemia, consequently [25]. Additionally, CVD is not an absolute contraindication to PA, however, patient with CVD should undergo a supervised cardiac rehabilitation program first, complete investigations and obtain cardiology clearance to be able to participate in PA program [26].

According to the risk level, 16.7 % of the high-risk referrals were directly referred to the wellness gym instead of the healthy lifestyle clinic, while 26 % of the moderate-risk referrals had direct wellness gym referrals lacking wellness champion review. The American Heart Association recommends the use of cost-effective screening procedures to identify at-risk individuals who should be medically evaluated before initiating an exercise program [27]. While most studies [[28], [29], [30]] indicate a reduction in cardiovascular events with increasing regular physical activity, several studies [31,32] signify a range between 4.4 % and 13.6 % of acute myocardial infarction associated with exertion. Evidence indicates that strenuous physical activities might mimic cardiac maladaptation including accelerated coronary artery calcification, the release of exercise-induced cardiac biomarkers, myocardial fibrosis, and increased risk of atrial fibrillation [33]. Data on moderate exercise and the risk of myocardial infarction is limited. One case-crossover study within the Myocardial Infarction Registry in Augsburg, Germany, reported a modest increase in the risk of myocardial infarction within 2 h of participation in moderate exertion (METs = 5; RR, 1.6 [95 % CI, 1.2−2.1]) [34].

No observed changes in the overall rate of inappropriate referrals between pre and post interventions, however, there was a marked reduction in referrals with no “Exercise Wellness Gym Assessment Form”, the first and most important area that ensure risk level is computed. Reasons could be related to referral process complexity, information transfer, lack of feedback system and work overload. A systematic review of intervention studies to improve referral appropriateness showed that peer review and feedback has been more successful in improving referral appropriateness when compared to individual-level interventions [35]. Factors related to physicians, patients and doctor-patient relationship are amongst the influential factors. In agreement to our study, Tobin-Schnittger et al. in their work, indicated that mixed interventions combining referral guidelines, templates and feedback have improved the referral quality [36].

## 5 Limitations, strengths, and implications

The study has several limitations that need to be considered. First, the data are derived from a single wellness center in Qatar, and the findings may not be generalizable to other settings or populations. In addition, the unique characteristics of the healthcare system in Qatar and the specific context of the wellness services in primary healthcare settings may limit the external validity of the study. Second, the retrospective nature of the analysis poses inherent limitations. Third, the study's focus on quantitative data may not capture the full complexity of the factors influencing inappropriate referrals. Qualitative insights from healthcare providers and patients could provide a more comprehensive understanding of the barriers and facilitators to the referral process. Fourth, the interventions implemented during the study period may have overlapping effects, making it challenging to isolate the impact of each intervention. A more controlled experimental design with distinct intervention phases could enhance the study's ability to attribute changes in referral patterns to specific interventions. Despite these limitations, the study contributes valuable insights into the challenges of the referral process to wellness services in a primary healthcare context. It addresses an important area, given the scarcity of studies on this topic both globally and locally, indicating a substantial gap in the existing literature. The utilization of electronic medical records provides a comprehensive dataset for analysis, allowing for a detailed examination of referral characteristics and trends over time. The multi-level interventions implemented demonstrate a proactive approach to addressing identified challenges in the referral system. The inclusion of training programs, feedback mechanisms, and the establishment of communication channels between healthcare providers and wellness team members reflects a comprehensive strategy to improve the appropriateness of referrals. The study's focus on evaluating the impact of interventions through a pre-post analysis adds strength to its design. Future research in this area could focus on several directions to further enhance our understanding and improve practices related to referrals to wellness services in primary care settings. Firstly, conducting qualitative research to explore the perspectives of healthcare providers, patients, and wellness team members could provide valuable insights into the factors influencing referral decisions and the impact of interventions. This could help in identifying additional strategies to improve the appropriateness of referrals. Secondly, longitudinal studies could be conducted to assess the long-term effects of interventions on referral patterns and patient outcomes. This could help in determining the sustainability of intervention strategies over time. Additionally, comparative studies across different healthcare settings and regions could help in identifying best practices and strategies that are effective in diverse contexts. Finally, exploring the role of technology, such as decision support tools or telehealth services, in improving the referral process could be an area for future research.

In Qatar, over half of the population (56 %) are not meeting the recommended level of PA (of at least 30 min of moderate PA for five days a week) [37]. Physical inactivity is a leading risk factor for noncommunicable disease and now identified as the fourth leading risk factor for global mortality [38]. Efforts should be made to provide access to resources that promote healthy behaviors and facilitate participation in an exercise program to improve outcomes. One increasingly common method for promoting PA in primary care setting is through ‘exercise referral schemes’ (ERS) [39]. However, health professionals cited barriers such as lack of time for proper consultation, hence, appropriateness of referral [40]. Our data suggest that completeness and timeliness of wellness templates as well as selecting the correct referral pathway based on risk level must be carefully crafted and implemented in order to improve referrals and timely provision of appropriate care. Inappropriate referrals to Wellness facility delay service utilization, lose patient track, and patient dissatisfaction.

## 6 Conclusion

This study, part of a quality improvement project using the Plan-Do-Study-Act (PDSA) cycle, provides valuable insights into the complexities of the wellness services referral process. It highlights a considerable rate of inappropriate referrals, indicating the need for a closer examination to address this issue. Despite that the interventions studied not showing a significant reduction in inappropriate referrals, this underscores the ongoing need for continuous improvement strategies.

Future research should focus on evaluating the effectiveness of more targeted and comprehensive interventions, such as face-to-face training to address root causes, the development of organizational guidelines, improved communication strategies, feedback mechanisms, and the establishment of a continuous monitoring system. These approaches could enhance the appropriateness of referrals and contribute to long-term gains in the referral process.

## Ethics approval and consent statement

All data and methods were carried out in accordance with relevant guidelines and regulations. Review and/or approval by an ethics committee was not needed for this study because it was part of a Quality Improvement Project aimed to improve the referral process and supervised by the Quality and Patient Safety Directorate, Primary Health Care Corporation (PHCC). Informed consent was not required for this study because of the retrospective nature of referral data extracted from medical records.

## Funding

This research received no specific grant from any funding agency in the public, commercial or profit sectors. Open access funding provided by 10.13039/100019779Qatar National Library.

## Data availability

The authors declare that the data supporting the findings of this study are available within the paper, a request for more detailed data should be sent to the corresponding authors with the permission of all authors.

## CRediT authorship contribution statement

**Sarah Musa:** Writing – review & editing, Writing – original draft, Validation, Supervision, Resources, Methodology, Investigation, Data curation, Conceptualization. **Sami Abdeen:** Writing – review & editing, Writing – original draft, Validation, Methodology, Investigation, Formal analysis, Data curation, Conceptualization.

## Declaration of competing interest

The authors declare that they have no known competing financial interests or personal relationships that could have appeared to influence the work reported in this paper.
