# Supplementary material for: The nature of inappropriate referrals to wellness services in primary care setting in Qatar: Outcome of multifaced interventions on rate and quality of referrals
Source: Heliyon. 2024 May 16;10(10):e31356. doi: 10.1016/j.heliyon.2024.e31356 (PMC11137418; doi:10.1016/j.heliyon.2024.e31356)
Supplement: Multimedia component 1 [file mmc1.docx]

Supplementary materials:

Supplementary Figure 1. Inappropriate referrals to wellness services per month (July 2022-August 2023, n=966)
